# Smart agriculture dataset in a tomato cultivation under different irrigation regimes

**DOI:** 10.1016/j.dib.2025.111521

**Published:** 2025-03-27

**Authors:** Laura Belli, Luca Davoli, Giulia Oddi, Luca Preite, Martina Galaverni, Tommaso Ganino, Gianluigi Ferrari

**Affiliations:** a Internet of Things (IoT) Lab, Department of Engineering and Architecture, University of Parma, 43124 Parma, Italy; b Department of Systems Engineering and Industrial Technologies, University of Parma, 43124 Parma, Italy; c Crop and Plant Science (Cro.P.S.) Lab, Department of Food and Drug, University of Parma, 43124 Parma, Italy

**Keywords:** Internet of things, Sensors, Agricultural irrigation, Tomato, Water, Soil, Air, Crop, GDD, Heat Units

## Abstract

This dataset contains data collected in a tomato cultivation (namely, a Solanum lycopersicum L. cv. HEINZ 1301 cultivation) located at the “Azienda Sperimentale Stuard,” Parma, Italy, through an IoT infrastructure featuring Long Range Wide Area Network (LoRaWAN)-enabled commercial devices deployed in the crop during the summer 2023 period (June 29–September 13). The IoT architecture also controls the irrigation system deployed to manage the watering conditions in the tomato crop, in detail considering three different experimental lines (each one associated with a different irrigation regime): (i) line #1 was irrigated with a water quantity equal to the irrigation recommendation provided by a national cloud service, denoted as Irriframe and developed by the Water Boards Italian Association (ANBI); (ii) line #2 was irrigated with a water quantity equal to 60% of line #1; (iii) line #3 was irrigated with a water quantity equal to 30% of line #1. The dataset comprises 4 different CSV files. The first three files (named as “stuard_environmental_data.csv,” “stuard_water_meter_data.csv,” and “stuard_soil_data.csv”) contain the information sampled every 10 minute by the IoT devices deployed in the crop—one environmental sensor, three water meters, and three soil sensors. The fourth CSV file (named as “indicators.csv”) contains the values of agronomic indicators of interest, calculated daily and mainly depending on daily air temperature values: (i) the Growing Degree Days (GDD) index and (ii) the Heat Units indicators, both calculated on the collected experimental tomato crop data.

Specifications Table

**Table:** 

Subject	Agriculture Engineering
Specific subject area	*Dataset collected through IoT devices (namely: soil, environmental, and water meter sensors) deployed in a tomato crop in Parma, Italy, in summer 2023*
Type of data	Raw, Analyzed
Data collection	Data was collected using the following LoRaWAN-enabled commercial devices. One Milesight EM500 CO2 environmental sensor measuring air moisture (%RH), temperature (°C), CO_2_ level (ppm), barometric pressure (hPa), and battery energy level (%). Three Talkpool OY1310 Water Meters sensors (one for each crop line) measuring water volume (m^3^). Three Milesight EM500 SMTC soil sensors, positioned at a 20 cm depth in the field (one for each crop line) and measuring soil electrical conductivity (µS/cm), soil moisture (%RH), and soil temperature (°C). To guarantee the LoRaWAN connectivity, a Milesight UG67 outdoor gateway was deployed in the main building of the “Azienda Sperimentale Stuard” farm.
Data source location	The experimental tomato crop was in the “Azienda Sperimentale Stuard,” Parma, Italy. The geographical coordinates in terms of (latitude, longitude) of the deployed LoRaWAN-enabled IoT devices are the following: - Milesight EM500 CO2: (44° 48’ 33.703’’ N, 10° 16’ 23.404’’ E) - Talkpool OY1310 water meters: ○ line#1: (44° 48’ 31.223’’ N, 10° 16’ 22.612’’ E) ○ line #2: (44° 48’ 31.208’’ N, 10° 16’ 22.756’’ E) ○ line #3: (44° 48’ 31.201’’ N, 10° 16’ 22.893’’ E) - Milesight EM500 SMTC devices: ○ line #1: (44° 48’ 31.424’’ N, 10° 16’ 22.673’’ E) ○ line #2: (44° 48’ 31.414’’ N, 10° 16’ 22.817’’ E) ○ line #3: (44° 48’ 31.403’’ N, 10° 16’ 22.961’’ E)
Data accessibility	Mendeley data repository name: “IoT-based Data Collection in a Tomato Cultivation Under Different Irrigation Regimes” Data identification number: doi: 10.17632/35wh56287y.2 Direct URL to data: https://doi.org/10.17632/35wh56287y.2 Mendeley URL to data: https://data.mendeley.com/datasets/35wh56287y/2
Related research article	Galaverni, M., Oddi, G., Preite, L., Belli, L., Davoli, L., Marchioni, I., Rodolfi, M., Solari, F., Beghè, D., T., Vignali, G., & Ferrari, G. (2025). An IoT-based data analysis system: A case study on tomato cultivation under different irrigation regimes. Computers and Electronics in Agriculture, vol. 229, 2025, doi: 10.1016/j.compag.2024.109660.

## 1 Value of the Data

• The experimental data, collected in a real tomato crop in the northern of Italy, can be used to estimate and evaluate the trends of soil and environmental parameters during the day, considering the three different irrigation regimes applied.
• Researchers can re-use the data contained in the dataset combining and comparing them with the information collected in similar crops located in different geographical areas, to estimate the variability of soil and environmental parameters.
• Data can be analyzed and processed with statistical techniques, as well as through algorithms based on Machine Learning (ML) and Deep Learning (DL) models, considering both single data sources and a combination of those available.
• The scientific community can leverage this dataset to define agronomic indicators of interest, based on calculated parameters (e.g., GDD and Heat Units).
• Researchers can use this dataset to model and simulate, with realistic values, an outdoor irrigation system for agriculture activities.

## 2 Background

This dataset [1] has been generated in the aim of a Smart Agriculture (SA) research activity looking at monitoring the tomato crop response to different irrigation regimes, through the implementation of an IoT LoRaWAN-enabled monitoring system. More in detail, the tomato irrigation has been scheduled according to the watering recommendations provided by an Italian platform, denoted as “Irriframe”, which is, a national cloud service developed by the Water Boards Italian Association (“*Associazione Nazionale Consorzi di gestione e tutela del territorio e acque irrigue*,” ANBI) [2] to provide guidelines for efficient water resource utilization in the agricultural sector. In particular, Irriframe suggests the amount of water to be supplied to the crops and the corresponding irrigation timing. Thus, the experimentation was conducted on tomatoes grown at three different irrigation regimes, namely, applying the 100%, 60%, and 30% of the Irriframe provided recommendation.

## 3 Data Description

The dataset is composed of four CSV files. Three of these files contain the information generated by the different enrolled IoT devices (namely: environmental sensor, water meters, and soil sensors) and the environmental data are sampled every 10 minutes. To this end, relevant parameters are: timestamp; IoT device identifier; air moisture and temperature; carbon dioxide (CO_2_) level; barometric pressure; battery percentage; tomato line identifier; water volume; soil electrical conductivity, moisture, and temperature. The fourth CSV file contains daily values of agronomic indicators, mainly based on the average daily air temperature values, such as: the daily values of Growing Degree Days (GDD) and Heat Units (namely: standard day degree; daily mean temperature; daily maximum temperature above *T*_base_; daily maximum temperature; daily maximum temperature above *T*_base_ with reduction of *T*_cutoff_; Ontario units).

In the following, a detailed description of each file column is provided (Table 1, Table 2, Table 3, Table 4).

**Table 1:** CSV file ``stuard_environmental_data.csv''.

Column	Column Name	Description	Measure Unit
0	id	A unique integer identifier of the row	-
1	device_identifier	A unique 64 characters identifier of the IoT device	-
2	ts_generation	Data sampling time instant	ms
3	co2 (Fig. 1)	CO_2_ concentration level Range: 400 - 5000 ppm Accuracy: ± (30 ppm + 3% of reading) (0°C - 50°C, 0 - 85%RH) Resolution: 1 ppm	ppm
4	humidity (Fig. 2)	Air moisture level Range: 0% to 100% RH Accuracy: 10% to 90% RH (±3%), below 10% and above 90% RH (±5%) Resolution: 0.5% RH	%RH
5	pressure (Fig. 3)	Barometric Pressure Range: 300 - 1100 hPa (-40°C - 85°C) Accuracy: ±1 hPa Resolution: 0.1 hPa	hPa
6	temperature (Fig. 4)	Air temperature Range: -30°C to + 70°C Accuracy: 0°C to + 70°C (±0.3°C), -30°C to 0°C (±0.6°C) Resolution: 0.1°C	°C
7	battery	Device battery level Range: 0% to 100%	%

**Fig. 1:**
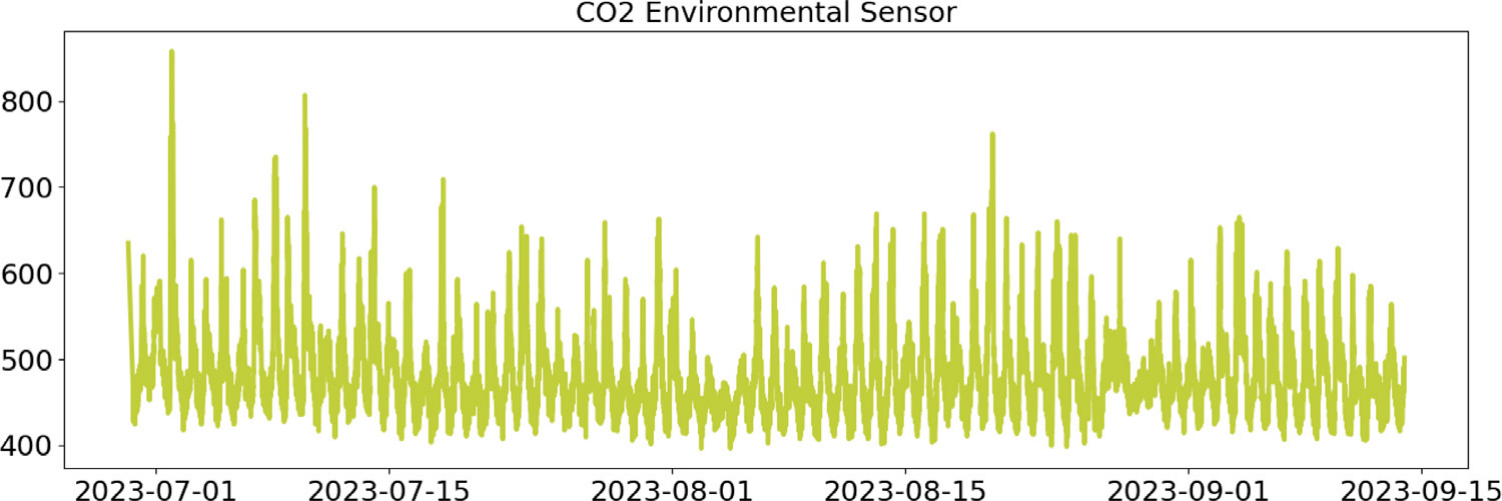
Air CO_2_ concentration (ppm) level sampled by the environmental sensor.

**Fig. 2:**
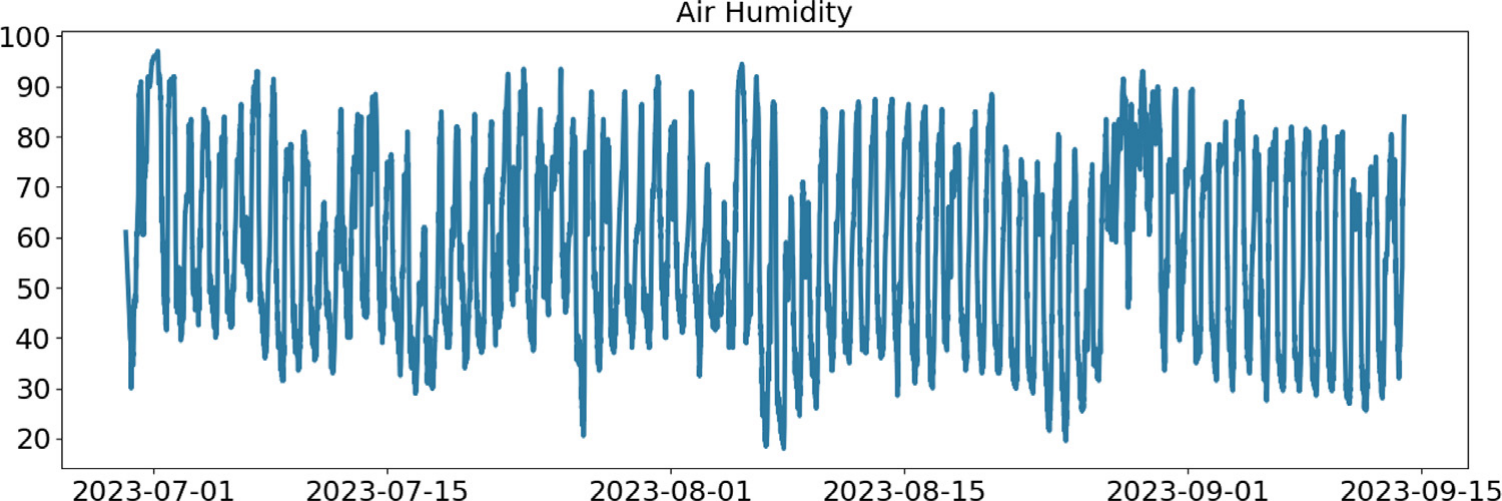
Air humidity (%RH) level sampled by the environmental sensor.

**Fig. 3:**
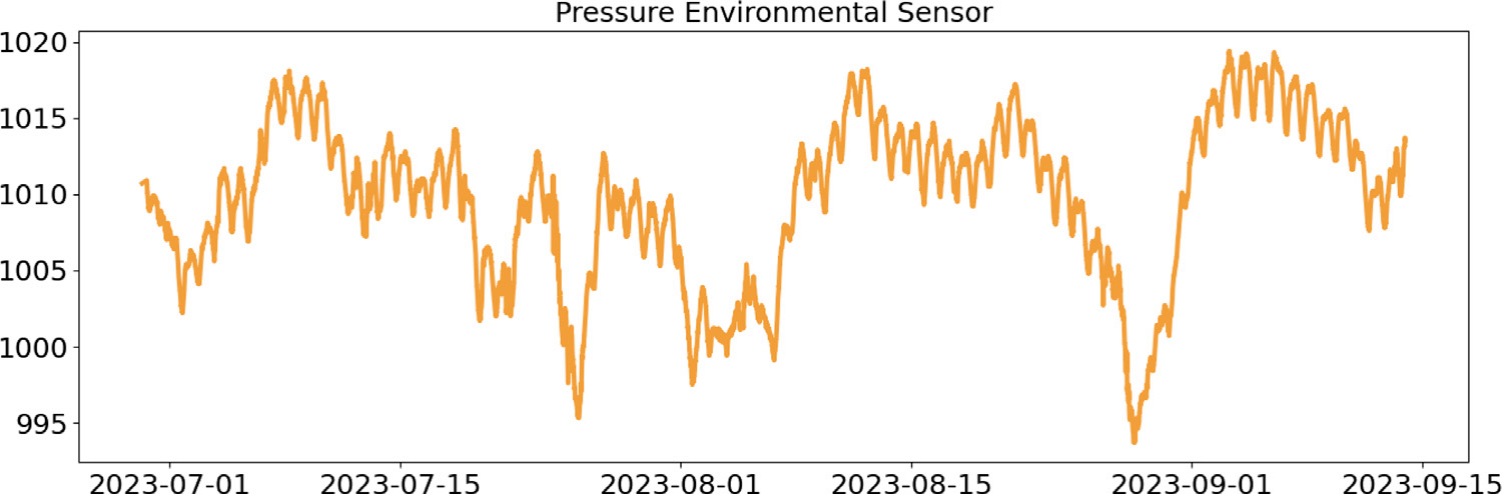
Air barometric pressure (hPa) sampled by the environmental sensor.

**Fig. 4:**
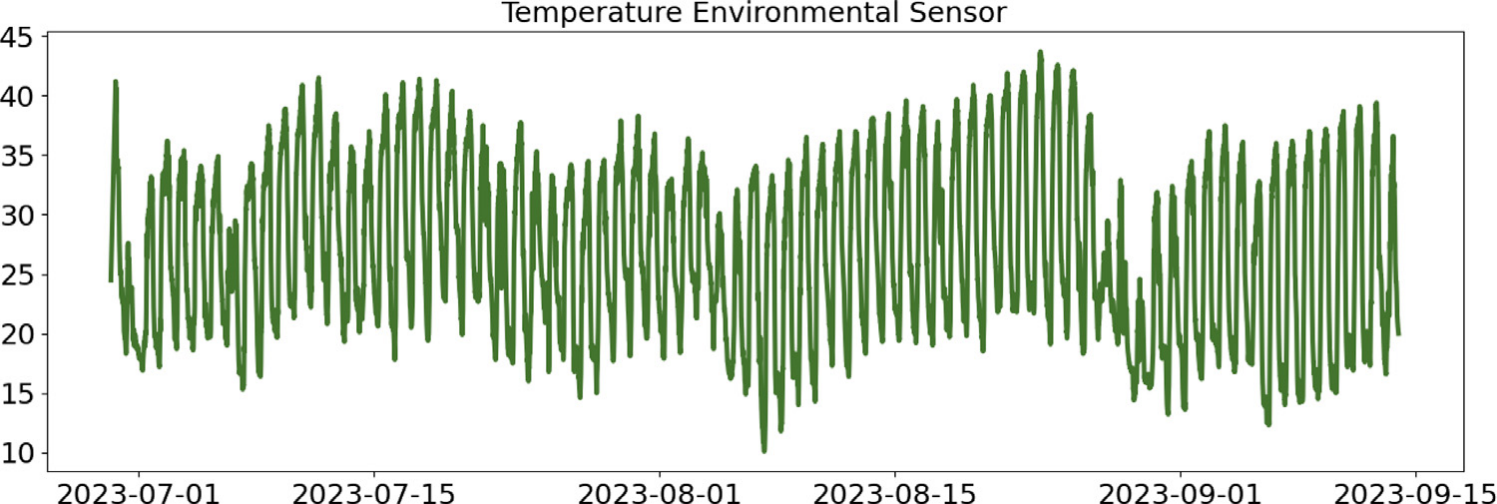
Air temperature (°C) sampled by the environmental sensor.

**Table 2:** CSV file ``stuard_water_meter_data.csv''.

Column	Column Name	Description	Measure Unit
0	id	A unique integer identifier of the row	-
1	ts_generation	Data sampling time instant	ms
2	device_identifier	A unique 64 characters identifier of the IoT device	-
3	line	Integer value representing the tomato line where the device is installed. Range: values in [1,2,3]	-
4	current_volume (Fig. 5)	Water volume	m^3^

**Table 3:** CSV file ``stuard_soil_data.csv''.

Column	Column Name	Description	Measure Unit
0	id	A unique integer identifier of the row	-
1	ts_generation	Data sampling time instant	ms
2	device_identifier	A unique 64 characters identifier of the IoT device	-
3	line	Integer value representing the tomato line where the device is installed Values in: [1,2,3]	-
4	electrical_conductivity (Fig. 6, Fig. 7, and Fig. 8)	Soil electrical conductivity	µS/cm
5	humidity (Fig. 9, Fig. 10, and Fig. 11)	Soil moisture level Range: 0% - 100% RH Accuracy: ±2% (0∼50%), ±3% (50%∼100%) Resolution: 0.01%	%RH
6	temperature (Fig. 12, Fig. 13, and Fig. 14)	Soil temperature Range: -40°C - 80°C Accuracy: ±0.5°C Resolution: 0.1°C	°C
7	battery	Device battery level Range: 0% to 100%	%

**Table 4:** CSV file ``indicators.csv''.

Column	Column Name	Description	Measure Unit
0	ts_generation	Indicator generation time instant	ms
1	gdd (Fig. 15)	GDD cumulation (starting from 01/01/23) Range: [13, 905.4]	°C
2	standard_day_degree (Fig. 15)	Calculated value of the standard day degree	°C
3	daily_mean_temperature (Fig. 15)	Calculated value of the daily mean temperature	°C
4	daily_max_above_Tbase (Fig. 15)	Calculated value of the daily maximum temperature above *T*_base_	°C
5	daily_max (Fig. 15)	Calculated value of the daily maximum temperature	°C
6	daily_max_reduction (Fig. 15)	Calculated value of the daily maximum temperature above *T*_base_ with a reduction of *T*_cutoff_	°C
7	ontario_units (Fig. 15)	Calculated value of the daily Ontario unit	°C

**Fig. 6:**
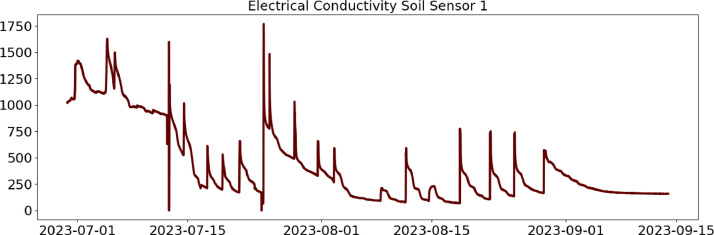
Soil electrical conductivity (µS/cm) sampled by the soil sensor deployed in the tomato line #1.

**Fig. 7:**
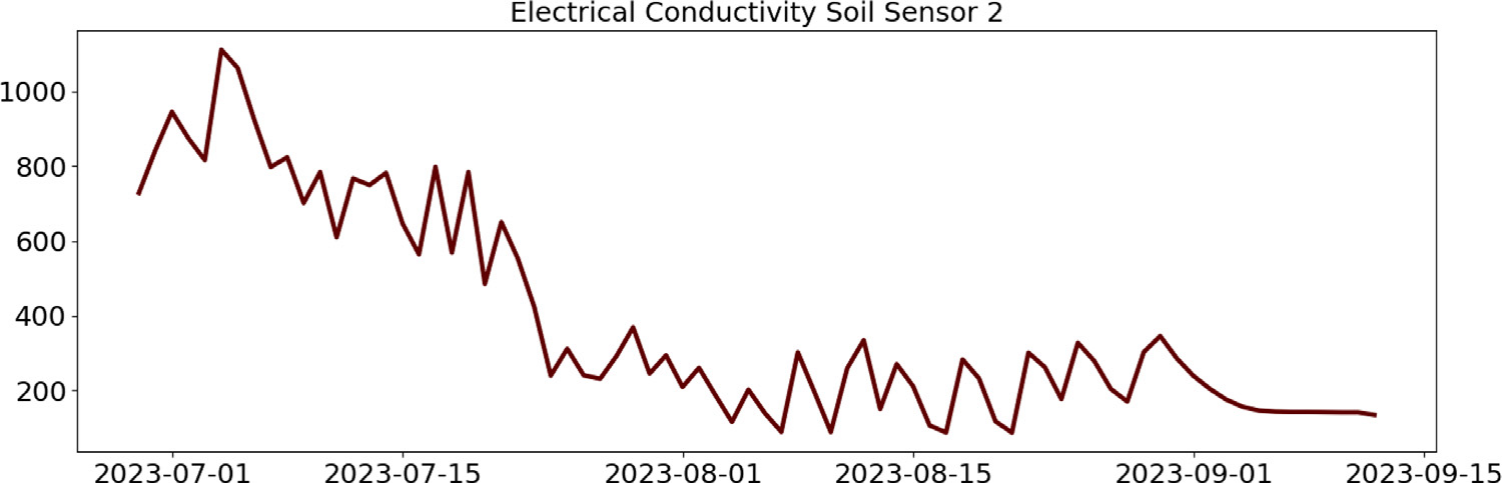
Soil electrical conductivity (µS/cm) sampled by the soil sensor deployed in the tomato line #2.

**Fig. 8:**
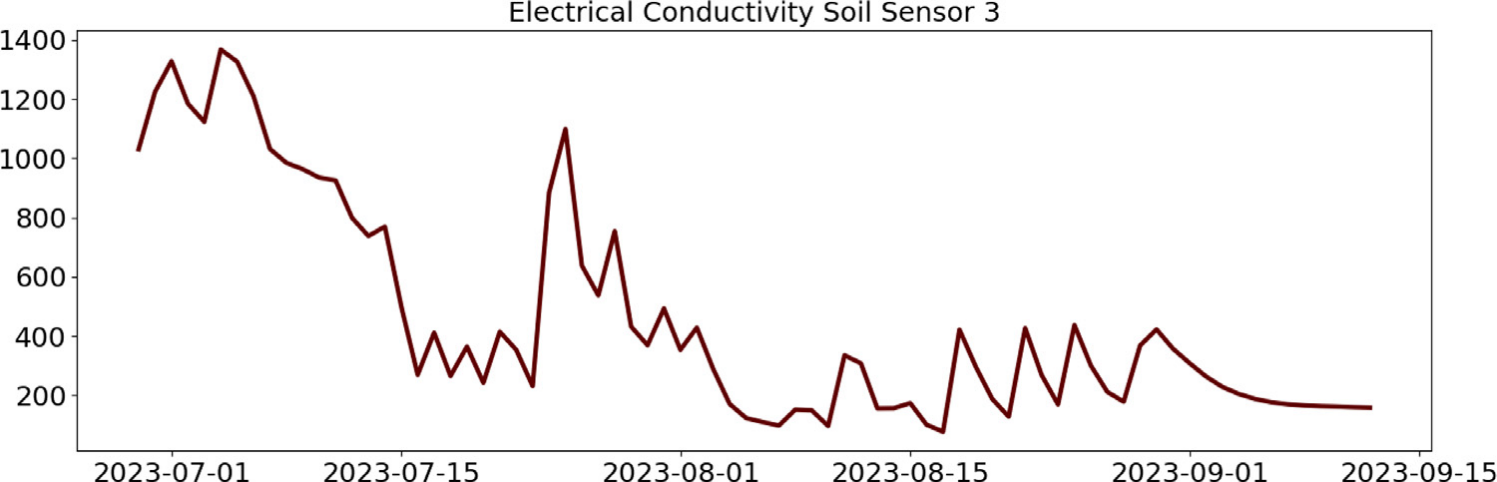
Soil electrical conductivity (µS/cm) sampled by the soil sensor deployed in the tomato line #3.

**Fig. 9:**
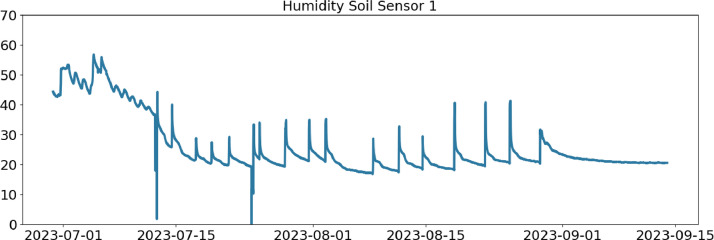
Soil humidity (%RH) sampled by the soil sensor deployed in the tomato line #1.

**Fig. 10:**
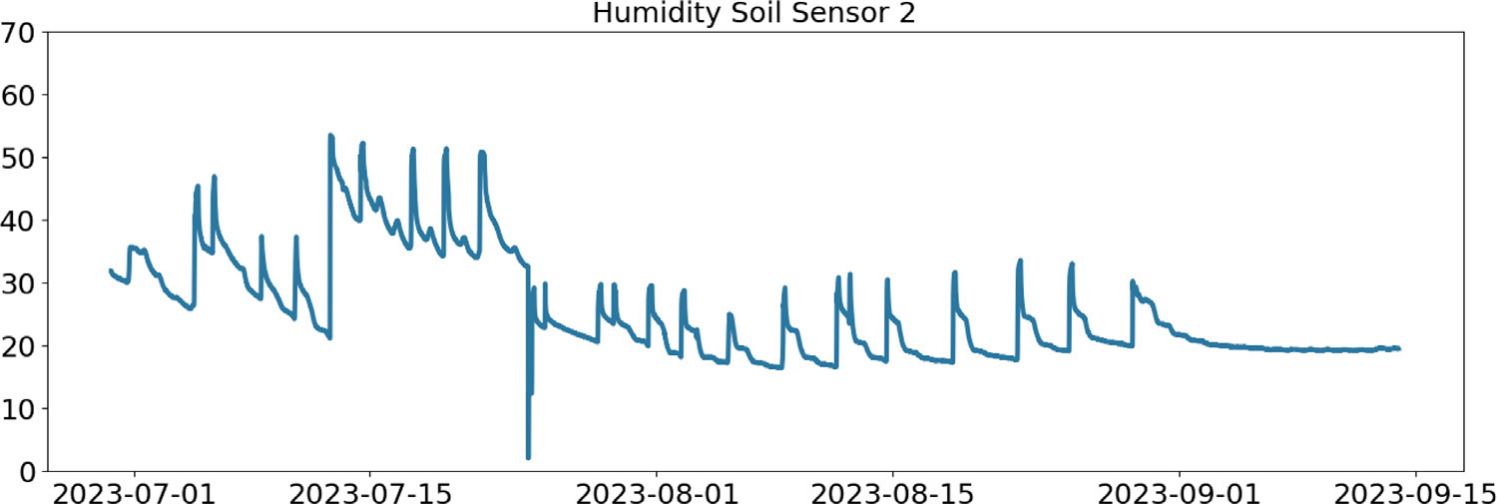
Soil humidity (%RH) sampled by the soil sensor deployed in the tomato line #2.

**Fig. 11:**
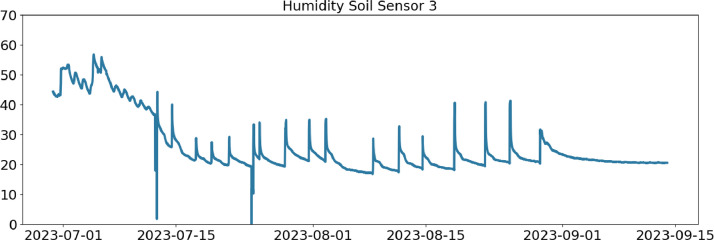
Soil Humidity (%RH) Level Soil Sensor deployed in the tomato line #3.

**Fig. 12:**
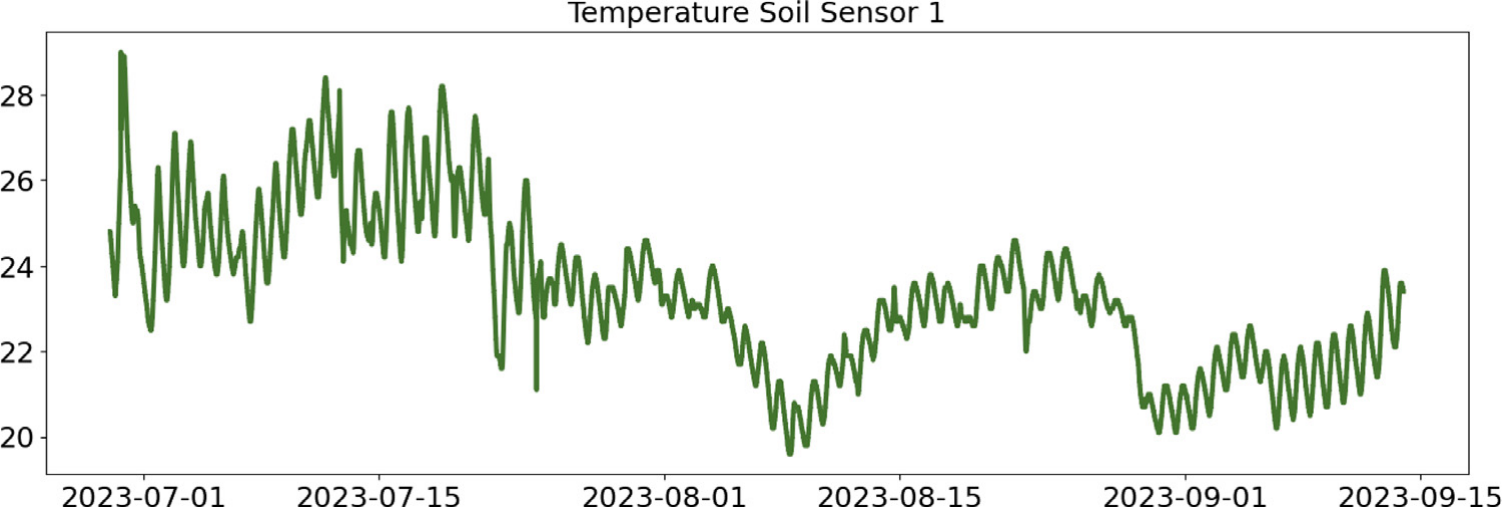
Soil temperature (°C) sampled by the soil sensor deployed in the tomato line #1.

**Fig. 13:**
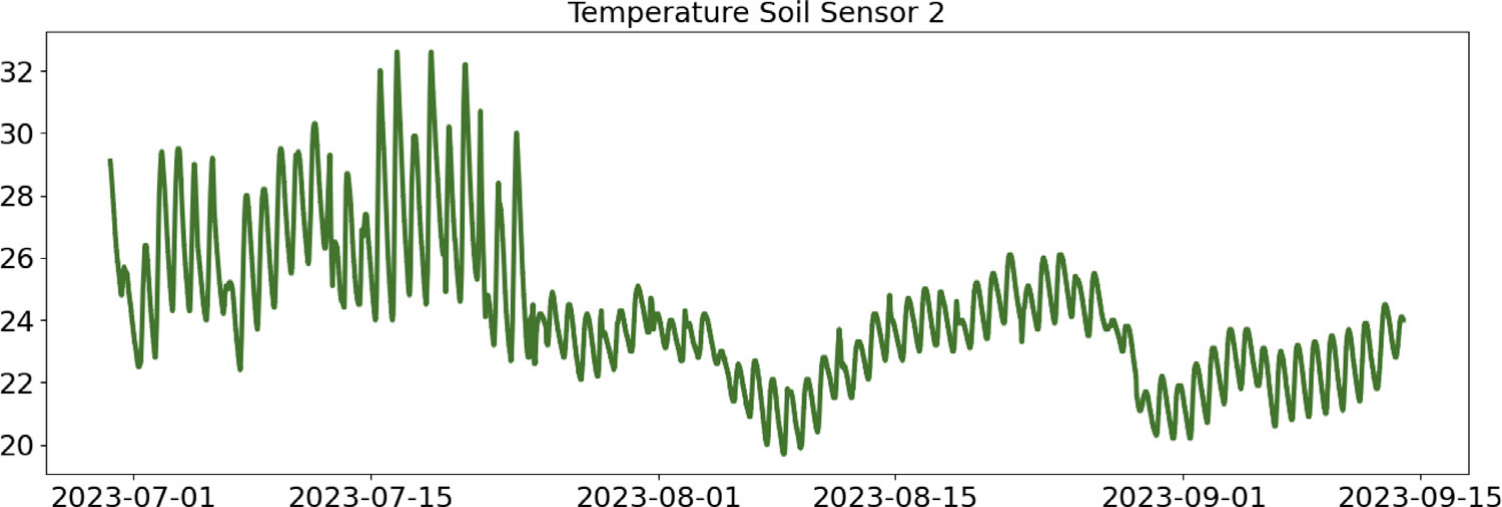
Soil temperature (°C) sampled by the soil sensor deployed in the tomato line #2.

**Fig. 14:**
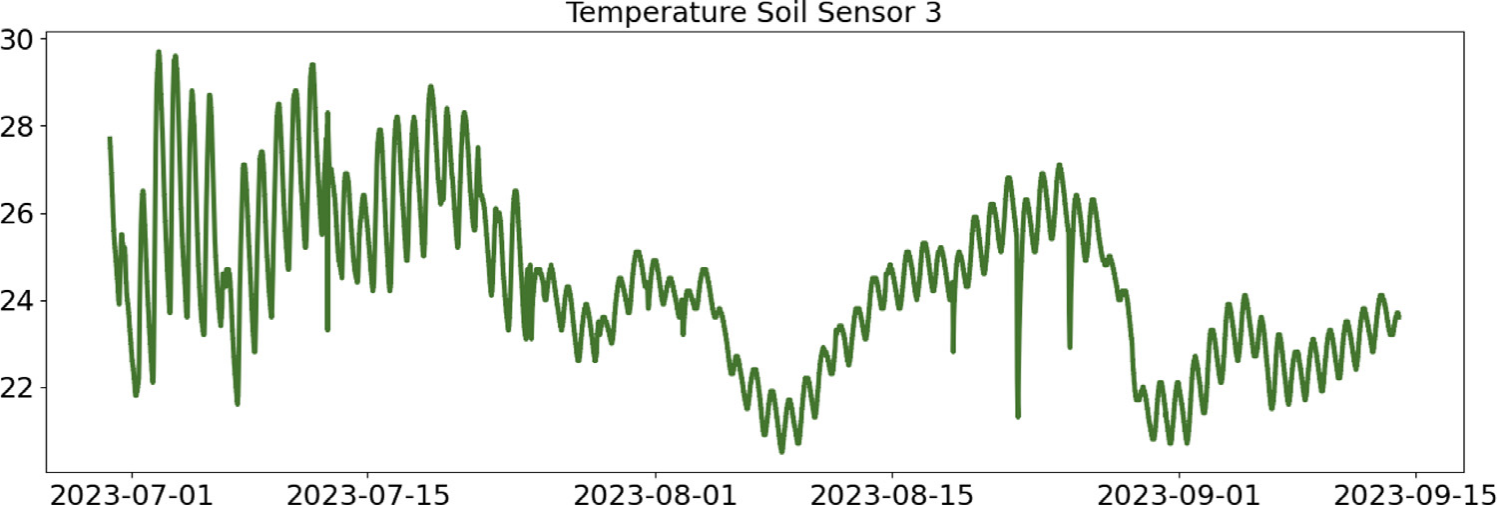
Soil temperature (°C) sampled by the soil sensor deployed in the tomato line #3.

**Fig. 15:**
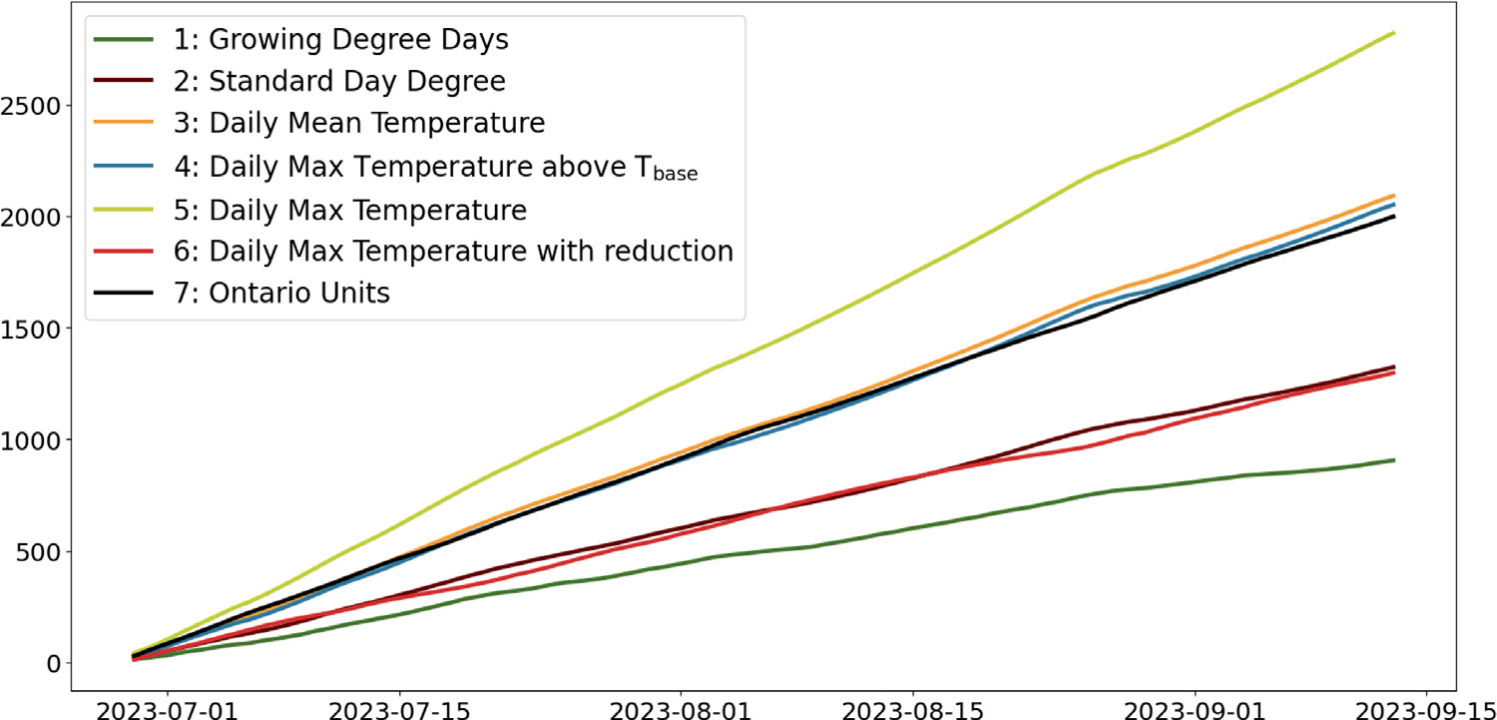
Calculated agronomic indicators, namely: (1) Growing Degree Days (GDD); (2) Standard Day Degree; (3) Daily Mean Temperature; (4) Daily Maximum Temperature; (5) Daily Maximum Temperature above T_base_; (6) Daily Maximum Temperature with reduction above T_base_ with a reduction of T_cutoff_; (7) Ontario Units.

## 4 Experimental Design, Materials and Methods

**Fig. 16:**
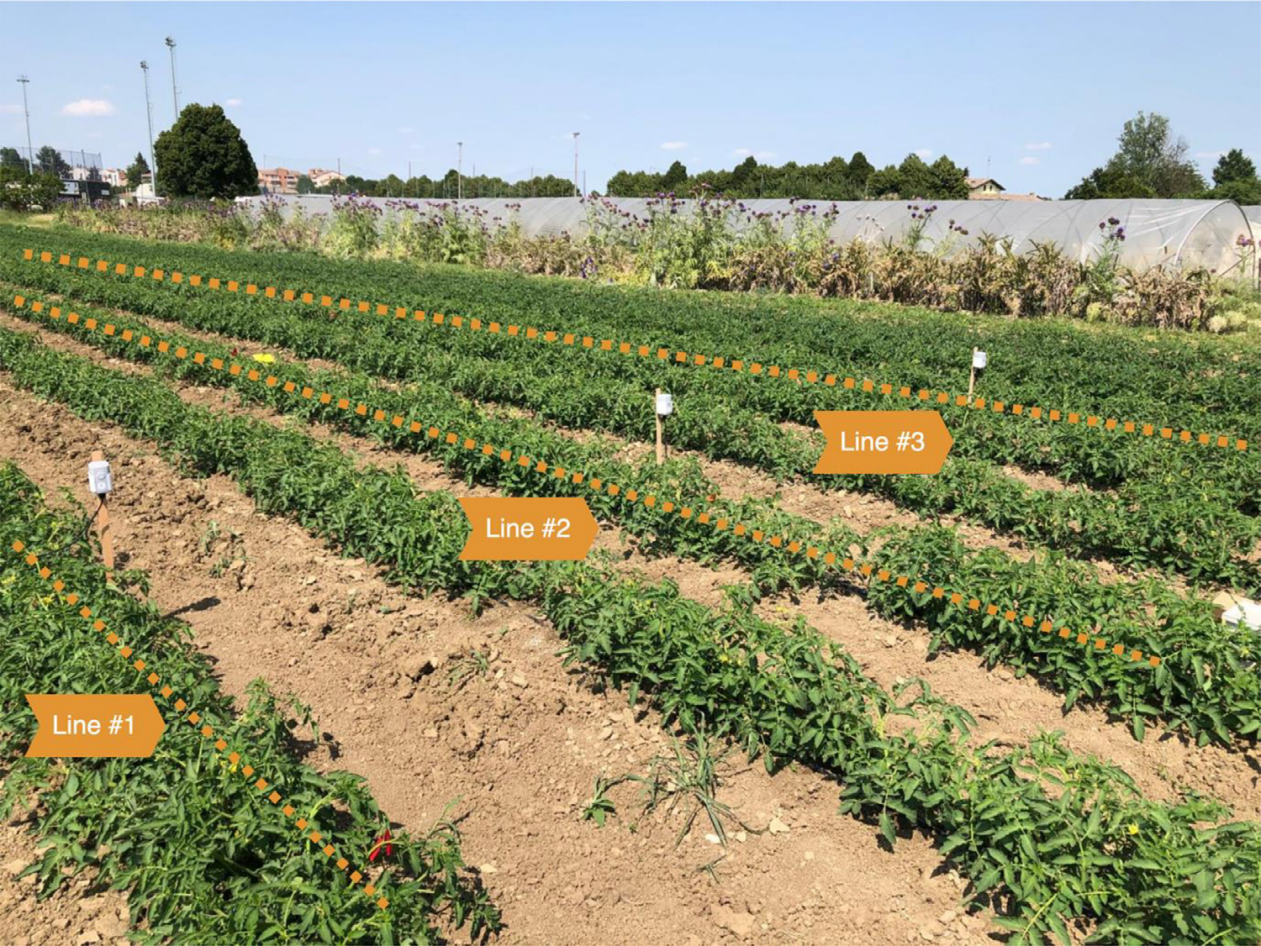
Experimental tomato crop area.

The agricultural information composing the dataset were collected during the summer period ranging from June 29, 2023, to September 13, 2023, through the deployment of several different LoRaWAN-enabled devices, as shown in Fig. 16.

The experimental activity involved a single tomato crop, with an irrigation system expedient to associate a different watering regime with three experimental tomato lines. In fact, the tomatoes irrigation strategy has been scheduled according to the watering recommendations provided by the Italian “Irriframe” platform, a national cloud-based service developed by the ANBI Association. Irriframe provides daily irrigation recommendations, in terms of irrigation volume and duration, on the basis of the environmental conditions, specific type of cultivation, and crop location, dimensions and irrigation system.

**Fig. 5:**
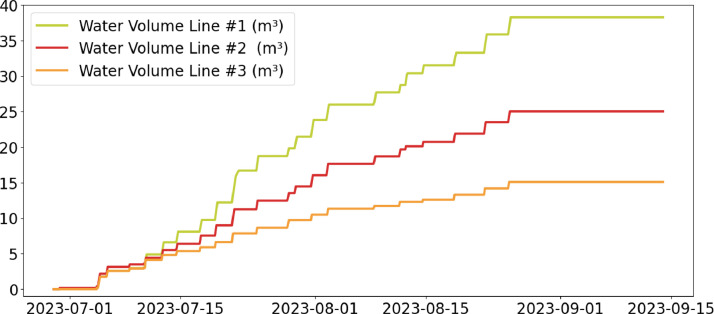
Water volumes (m^3^) sampled by the water meters in the tomato lines #1–#3.

During the experimentation, regular irrigations, characterized by a quantity of water equal to 100% (line #1), 60% (line #2), and 30% (line #3) of the recommendation provided by the Irriframe service, respectively, were applied by the farmer to the three experimental lines, starting at the beginning of the blooming phase. The amount of water provided to each line is shown in Fig. 5 and detailed in the CSV file ``stuard_water_meter_data.csv'' CSV file in the dataset.

Regarding crop fertilization, during the first three weeks after transplanting, 118.8, 90, and 180 units/ha of N, P, K, respectively, were distributed in the crop the farmer. During the crop season, additional amounts of N, P, K were added weekly, for a final amount of 180, 90 and 200 units/ha, respectively.

The IoT infrastructure enabling the continuous collection and monitoring of the crop has been deployed in the experimental agricultural area installing different LoRaWAN sensing devices in the three experimental rows. More in detail, the IoT architecture consists of a set of LoRaWAN devices communicating with a LoRaWAN Milesight UG67 outdoor gateway deployed in the farm's main building to allow data collection from the whole monitored area. All the deployed IoT devices operated as LoRaWAN Class A end nodes, were registered on the open source The Things Network (TTN) LoRaWAN platform [3], and were configured to transmit their collected information every 10 minutes. Then, inside the TTN platform, a webhook was configured to push all the incoming data toward a remote software platform, denoted as Agriware, developed by the Internet of Things (IoT) Lab of the University of Parma, Italy, and in charge of validating and persistently storing information in a MySQL-based relational database. The Agriware platform was also responsible for the calculation of daily values of agronomic indicators, through Python-based scripts.

The first agronomic indicator is the Growing Degree Days (GDD), and is based on: average daily air temperature, measurable through environmental sensors; the *base temperature* (*T*_base_), set to 10°C for the tomato plant; and *cutoff temperature* (*T*_cutoff_), set to 32°C for the tomato plant. Once these values are known, the daily GDD values can be calculated according to [4]. The other agronomic indicators, knows as Heat Units, are based on: average daily air temperature; *T*_base_; *T*_cutoff_. Heat Units are calculated daily using six different methods, as defined by [5]: (i) standard degree days; (ii) daily mean temperature; (iii) daily maximum temperature above *T*_base_; (iv) daily maximum temperature; (v) daily maximum temperature above T_base_ with reduction of *T*_cutoff_; (vi) Ontario unit. All the calculated agronomic temperature indicators are expressed in°C.

## Limitations

The dataset was collected over a limited period, covering the growing and harvesting phases of a tomato cultivation. Moreover, the sampling interval of 10 minutes was selected as a *trade-off* between monitoring requirements (to guarantee a sufficient accuracy) and LoRaWAN sensing and devices battery saving.

## Ethics Statement

The authors have read and follow the ethical requirements for publication in Data in Brief and confirm that the current work does not involve human subjects, animal experiments, or any data collected from social media platforms.

## CRediT Author Statement

**Laura Belli:** Conceptualization, Methodology, Software, Validation, Formal analysis, Investigation, Resources, Data curation, Writing – original draft, Writing – review & editing, Visualization. **Luca Davoli:** Conceptualization, Methodology, Software, Validation, Formal analysis, Investigation, Resources, Data curation, Writing – original draft, Writing – review & editing, Visualization. **Giulia Oddi:** Conceptualization, Methodology, Software, Validation, Formal analysis, Investigation, Resources, Data curation, Writing – original draft, Writing – review & editing, Visualization. **Luca Preite:** Conceptualization, Methodology, Software, Validation, Formal analysis, Investigation, Resources, Data curation, Writing – review & editing, Visualization. **Martina Galaverni:** Conceptualization, Methodology, Software, Validation, Formal analysis, Investigation, Resources, Data curation, Writing – review & editing, Visualization. **Tommaso Ganino:** Conceptualization, Methodology, Validation, Formal analysis, Resources, Writing – review & editing, Supervision, Project administration, Funding acquisition. **Gianluigi Ferrari:** Conceptualization, Methodology, Validation, Formal analysis, Resources, Writing – review & editing, Supervision, Project administration, Funding acquisition.

## Data Availability

Mendeley DataIoT-based Data Collection in a Tomato Cultivation Under Different Irrigation Regimes (Original data).
